# Abscence of specific humoral response in three dogs with clinical leishmaniosis

**DOI:** 10.1186/s13028-025-00814-9

**Published:** 2025-06-08

**Authors:** Sergio Villanueva-Saz, Diana Marteles, Ámparo Ortuñez, María C. Aceña, Janine E. Davies, Cristina Riera, María Borobia, Maite Verde, Álex Gómez

**Affiliations:** 1 Animal Pathology Department, Veterinary Faculty, Zaragoza University, C/ Miguel Servet 177, Zaragoza, 50013 Spain; 2 Veterinary Faculty, Instituto Agroalimentario de Aragón-IA2, Zaragoza University, C/ Miguel Servet 177, Zaragoza, 50013 Spain; 3 Clinical Immunology Laboratory, Veterinary Faculty, Zaragoza University, C/ Miguel Servet 177, Zaragoza, 50013 Spain; 4 La Ribera Veterinaris, C/ Fotja, Palma de Mallorca, 07610 Spain; 5 Veterinary Faculty, HV, C/ Miguel Servet 177, Zaragoza, 50013 Spain; 6 Departament de Biologia, Salut I Medi Ambient, Facultat de Farmacia, Universitat de Barcelona, Av. Joan XXIII 27-31, Barcelona, 08028 Spain

**Keywords:** Antibodies, Immune response, *Leishmania infantum*, Serology

## Abstract

**Background:**

Canine leishmaniosis, caused by *Leishmania infantum*, is a vector-borne disease. The immune response in infected dogs determines the clinical outcome, with a strong cell-mediated immune response linked to parasite control and mild clinical signs, while a humoral-dominant response is associated with severe disease. Low antibody levels in clinically asymptomatic dogs with negative molecular and/or parasitological test results may reflect prior exposure or the early stages of *Leishmania* infection. In contrast, elevated antibody levels are typically correlated with a high parasitic burden and active disease. The detection of dogs with clinical leishmaniosis and null-specific immune response against *L. infantum* is uncommon. However, this presentation has also been described in human leishmaniasis with the absence of humoral response detected by conventional serological methods.

**Case presentation:**

Case 1, a 9-year-old Border Collie, showed splenomegaly and *Leishmania* amastigotes within splenic macrophages. Case 2, a 10-month-old French Bulldog, had chronic anorexia and malabsorption syndrome with granulomatous splenitis and amastigotes confirmed by immunohistochemistry. Finally, case 3, a 7-year-old cross-breed, presented with cutaneous nodules and nasal ulcerative dermatitis, with *Leishmania* amastigotes detected histologically and confirmed by immunohistochemistry. All dogs were seronegative by two quantitative serological tests including indirect immunofluorescent test and enzyme-linked immunosorbent assay. The identification of the parasite in the affected organ established a clear cause-and-effect relationship. Consequently, anti-*Leishmania* treatment was initiated, consisting of allopurinol (10 mg/kg orally twice daily) and meglumine antimoniate (50 mg/kg subcutaneously twice daily for four weeks) in cases 1 and 3. In case 1, a favourable clinical response was noted, with a normal abdominal ultrasound and a negative result by quantitative molecular test from material obtained via ultrasound-guided splenic puncture. In case 3, the administration of meglumine antimoniate resulted in the resolution of dermatological signs. Clinical follow-up and anti-*Leishmania* treatment could not be performed for case 2.

**Conclusions:**

These findings highlight the diagnostic challenges in detecting clinical leishmaniosis in seronegative dogs. The absence of a specific humoral response should be considered, emphasizing the importance of using multiple diagnostic methods, including cytology, and histopathology with immunohistochemistry. This case series underscores the need for a comprehensive approach in diagnosing and managing canine leishmaniosis.

## Background

Canine leishmaniosis (CanL) is a zoonotic vector-borne disease caused by the protozoan *Leishmania infantum* in European Mediterranean countries, South and Central America, Africa and Central and Southwest Asia [1, 2]. The most common clinical signs of CanL are weight loss, anorexia, cutaneous lesions and lymphadenomegaly, followed by ocular lesions, mucous membrane ulceration, epistaxis and fever [2–4]. However, atypical lesions are also described affecting striated musculature, central nervous system, gonads and endocrine glands [5, 6]. The most frequent laboratory findings observed in CanL are nonregenerative anaemia, hyperproteinaemia, and a polyclonal gammopathy with low serum albumin: globulin ratio. Moreover, depending on the tissue affected, other biochemical parameters may vary; for instance, creatine kinase and lactate dehydrogenase levels may be elevated when skeletal muscle is involved [7, 8].

In Europe, serological methods including enzyme-linked immunosorbent assay (ELISA), indirect immunofluorescence antibody test (IFAT) and immunochromatographic rapid tests represent the most common methods used for detecting antibodies against *L. infantum* in infected dogs [9, 10]. In general, elevated *Leishmania* specific antibody levels in a single serum sample (three- to fourfold increase compared to a well-established laboratory reference cut-off) together with the presence of clinical signs and clinicopathological findings are linked to increased parasitism [11, 12].

Nevertheless, serological methods may yield suboptimal results in certain infected dogs. In cases of recent infection, serological tests may return negative results despite active infection, as the interval for seroconversion in naturally infected animals can range from 1 to 22 months. Additionally, the efficacy of serological tests may be influenced by factors such as poor standardization, high cost in some instances, and variability in sensitivity and specificity [3, 13]. The IFAT test is traditionally recommended by the World Organization for Animal Health (OIE) as the reference serological method due to its high sensitivity and specificity in numerous endemic regions. However, it is noteworthy that the specificity of this assay can be compromised by serological cross-reactions with other infections or metabolic disorders [14]. In all three cases reported in this study, an in-house quantitative ELISA test for anti-*Leishmania* antibodies, which demonstrated a sensitivity of 99.37% and a specificity of 97.50%, was performed [15].

Dogs with chronic *Leishmania*-induced cutaneous lesions, especially those presenting with a papular dermatitis together with asymptomatic infected dogs can be seronegative or show low levels of specific antibodies [16, 17]. Papular dermatitis due to *L. infantum* is associated with a parasite specific cell mediated immunity and a poor humoral immune response [16]. Therefore, complementary confirmatory techniques should be included, such as direct observation of *L. infantum* amastigotes by cytology, histopathology, or immunohistochemistry (IHC) and also molecular techniques like PCR [9, 18]. In this context, the identification of the parasite in the affected organ can clearly establish a cause–effect relationship [19]. Additionally, quantitative PCR (qPCR) is widely used for *Leishmania* DNA detection [20]. The aim of this case series was to report the absence of *L. infantum* humoral immune response in dogs with clinical leishmaniosis confirmed by direct observation by both cytological-histological examination and IHC.

## Case presentation

**Fig. 1:**
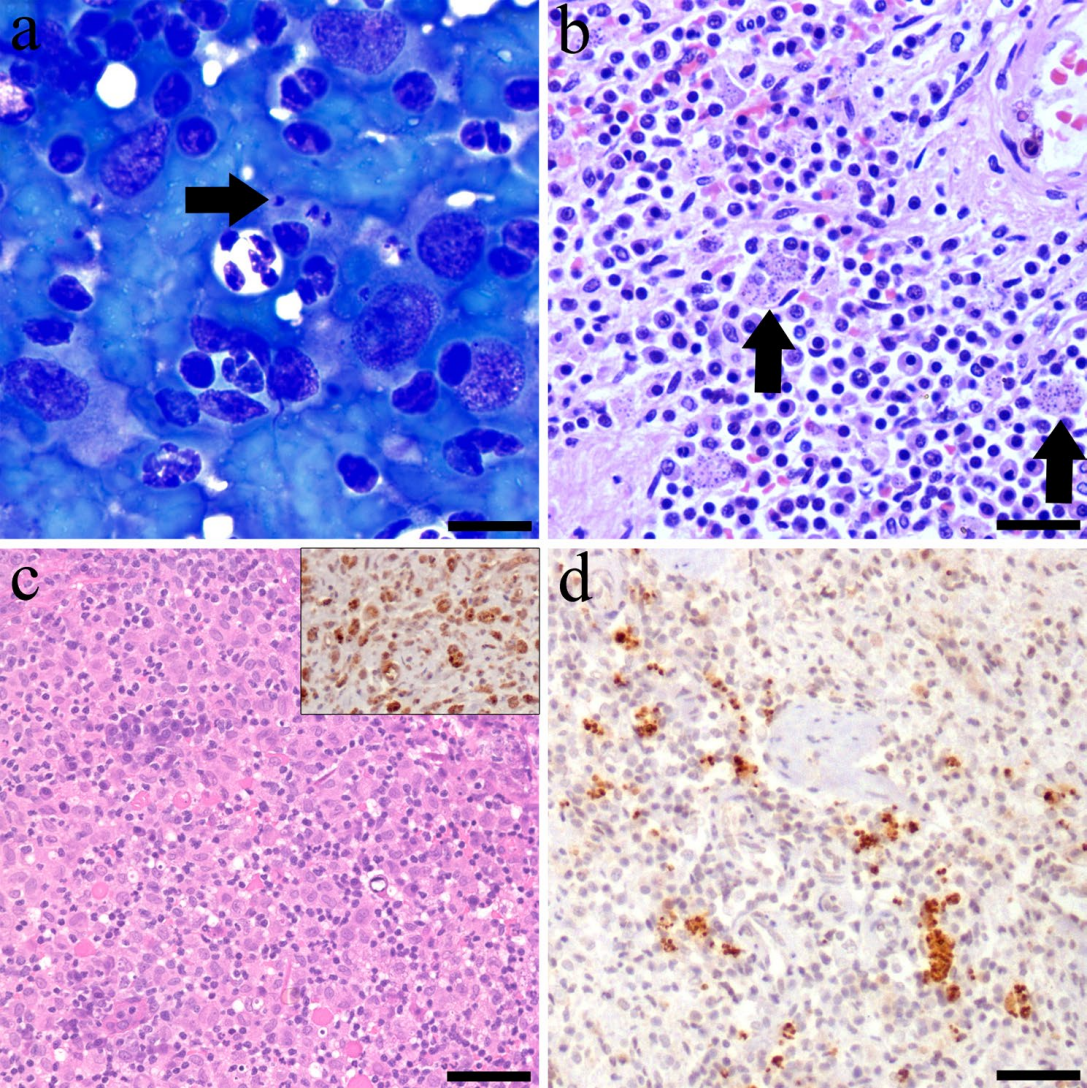
Diagnostic confirmation tests of the case series report. **(a)** Case 1: spleen cytology showed a group of macrophages containing within the cytoplasm forms compatible with *Leishmania* amastigotes, showing conspicuous kinetroplasts (arrow). Modified Wright stain; bar = 50 μm. **(b)** Case 2: splenic histology revealed multiple macrophages with cytoplasmic *Leishmania* amastigotes. Haematoxylin-eosin (H-E); bar = 200 μm. **(c)** Case 3: histology showed a severe pyogranulomatous dermatitis. H-E; bar = 300 μm. Inset: Numerous positive *L. infantum* amastigotes within the cytoplasm of dermal macrophages. *L. infantum* immunohistochemistry (IHC); bar = 100 μm. **(d)** Case 2: Macrophages with intracytoplasmic immunopositive *L. infantum* amastigotes are distributed throughout the spleen. *L. infantum* IHC; bar = 200 μm

Case study 1, a 9-year-old male Border Collie, that presented with marked apathy and lethargy. Complete blood count (CBC), serum biochemistry and serum protein electrophoresis were within normal parameters. Additionally, abdominal ultrasound (US) evaluation confirmed splenomegaly with diffuse coarsened echotexture and several mixed echoic lesions with irregular surface. A US-guided splenic puncture was obtained for laboratory analysis. Cytological examination revealed the presence of *Leishmania* amastigotes within the cytoplasm of macrophages (Fig. 1a).

Case study 2, a 10-month-old female French Bulldog, that presented with a history of chronic anorexia and malabsorption syndrome. CBC, serum biochemistry and serum protein electrophoresis revealed normochromic and normocytic anaemia (24–35; reference interval (RI): 37.3–61.7), low haemoglobin (12.3; RI: 13.1–20.5 g/dL), hypoproteinaemia (5.4; RI: 5.7–7.5 g/dL), hypoalbuminemia (1.1; RI 2.3–4.6 g/dL) and low albumin: globulin ratio (0.3; RI: 0.7–1.1). An abdominal US evaluation showed marked enlargement of the mesenteric lymph nodes and splenomegaly. An US-guided splenic puncture was obtained for laboratory analysis detecting moderate to low cellularity on a very bloody background, consisting of a lymphoid population with a predominance of small lymphocytes, hematopoietic cells, and neutrophils. Due to the absence of relevant findings, an incisional splenic biopsy was performed. Histopathology showed a severe, multifocal to coalescing, granulomatous splenitis with protozoal amastigotes consistent with *Leishmania* spp. within the cytoplasm of macrophages (Fig. 1b). IHC evaluation using an in-house rabbit polyclonal antibody specific for *L. infantum* (diluted 1:500), demonstrated the immunopositivity of intracytoplasmic *L. infantum* amastigotes in all three clinical cases (Fig. 1d) [21].

Case study 3, a 7-year-old male cross-breed dog, was presented with a history of abdominal multifocal cutaneous nodular masses (3 × 5 cm) and nasal ulcerative dermatitis that persisted over several months. During physical examination, a slight inflammation of the popliteal lymph nodes was also observed. CBC, serum biochemistry, serum protein electrophoresis and urine analysis were within the normal parameters. The cutaneous nodular masses were biopsied. Histologically, these nodules were composed of a high number of foamy macrophages and neutrophils, disrupting and expanding the dermis (Fig. 1c). The epidermis presented multifocal ulcers and sero-cellular crusts. Organisms compatible with *Leishmania* amastigotes were not observed histologically. However, IHC demonstrated cytoplasmic immunolabelling for *L. infantum* within macrophages (Fig. 1c).

To detect anti-*Leishmania* antibodies quantitative indirect in-house ELISA (sensitivity 99.37% and specificity 97.50%) were performed in all cases [15]. For the in-house ELISA, the crude antigen (strain MHOM/FR/78/LEM 75 belonging to *Leishmania infantum* zimodeme MON-1) was adjusted to a concentration of 20 µg/mL with sterile PBS. Briefly, each plate was coated with 100 µL/well of the 20 µg/mL antigen solution in 0.1 M carbonate/bicarbonate buffer (pH 9.6) and incubated overnight at 4 °C. Plates were then frozen and stored at − 20 ºC. One hundred microliters of dog serum diluted 1:800 in phosphate buffered saline (PBS) containing 0.05% Tween 20 (PBST) and 1% dry skimmed milk (PBST-M) were added to each well. The plates were incubated for 1 h (h) at 37 °C in a moist chamber. After washing the plates three times with PBST for 3 min (min) followed by one wash with PBS for 1 min, 100 µL of Protein A conjugated to horseradish peroxidase (Thermo Fisher Scientific, Waltham, Massachusetts, USA) diluted 1:20000 in PBST-M was added to each well. The plates were incubated for 1 h at 37 °C in a moist chamber, followed by washes with PBST and PBS as described above. The substrate solution (ortho-phenylene-diamine) and stable peroxide substrate buffer (Thermo Fisher Scientific, Waltham, Massachusetts, USA) were added (100 µL per well) and developed for 20 ± 5 min at room temperature in the dark. The reaction was terminated by adding 100 µL of 2.5 M H2SO4 to each well. Absorbance values were read at 492 nm (reference wavelength) in an automatic microELISA reader (ELISA Reader Labsystems Multiskan, Midland, Canada). Each plate included serum samples from a dog infected with *L. infantum* as confirmed by cytological examination as a positive control (calibrator) and serum samples from a healthy, noninfected dog from the blood donor program as a negative control. The same calibrator serum sample was used for all assays, and the plates with an interassay variation greater than 10% were discarded. All samples and controls were analysed in duplicate. The results were quantified as ELISA units (EU) compared to a positive control serum sample used as a calibrator that was arbitrarily set to 100 EU. Additionally, an in-house IFAT (sensitivity 99.36% and specificity 91.94%) was performed [15]. The IFAT was performed using promastigote forms of the strain MHOM/MON-1/LEM 75 zymodeme MON-1 as a whole-parasite antigen fixed on multi-spot slides (Thermo Fisher Scientific, Waltham, MA, USA). Sera from the dogs were assayed in serial twofold dilutions from 1:80 to 1:2560. Briefly, a twofold dilution of each serum was applied per well. The slides were incubated for 30 min at 37 °C in a moist chamber and then washed twice with PBS for 5 min and once more with distilled water. After the washing procedure, 20 µl of goat anti-dog IgG-fluorescein isothiocyanate conjugate (Sigma-Aldrich, Saint Louis, MO, USA) diluted 1:32 in 0.2% Evans blue was added to each well. The slides were incubated in a moist chamber at 37 °C for another 30 min in complete darkness and washed again as described above. After the second washing procedure, a few drops of mounting medium were placed on the cover slips. The slides were examined under a fluorescence microscope (Leica DM750 RH; Leica Microsystems, Wetzlar, Germany) at 400× magnification, and each well was compared to the fluorescence pattern seen in the positive and negative controls. Positive and negative controls were included on each slide. A positive control serum was obtained from a dog from Spain diagnosed with CanL, confirmed by a positive *L. infantum* isolation using a biphasic Novy, McNeal and Nicolle blood agar medium, and a negative control serum was obtained from a healthy, non-infected dog originating from a non-endemic area. Finally, For Western blot (WB) analysis (sensitivity 99.50% and specificity 98.75%, internal information), anti-*Leishmania* antibodies were detected using a whole *L. infantum* promastigote antigen (MHOM/FR/78/LEM75 zymodeme MON-1) [22, 23]. Antigen electrophoresis in 1% sodium dodecyl sulfate/15% polyacrylamide gels together with molecular mass protein standards (Standard Low Range; Bio-Rad, Hercules, CA, USA) was performed on a Mini-Gel AE 6400 Dual Mini Slab Kit (ATTO Corp., Tokyo, Japan). The gels were run at 100 V for 1 h at room temperature. Polypeptides were transblotted onto nitrocellulose sheets (0.45-mm pore size, HAWP 304 FO; Millipore Corp., Bedford, MA, USA), which were blocked with 20 mM Tris, 0.13 mM NaCl, pH 7.6 (TS) and 5% skimmed milk, overnight at 4 °C. The sheets were washed in TS and introduced into a multiscreen apparatus (Mini Protean II, Multiscreen Apparatus; Bio-Rad). Sera were diluted 1:200 in TS/1% skimmed milk and 0.2% Tween 20. Then 500 µl of each sample was introduced into each channel of the multiscreen apparatus and incubated for 2 h at 37 °C. Bound immunoglobulins were developed by incubation with a 1:1000 dilution of Protein A peroxidase conjugate (Thermo Fisher Scientific) for 1 h. After the sheets were washed three times with TST and a final time with TS, colour was developed with 4-chloro-1-naphthol (Thermo Fisher Scientific) and H2O2, and the reaction was stopped with tap water after 30 min. A serum sample was considered WB-positive if immunoreactivity against the 14 kDa and/or 16 kDa low-molecular-weight polypeptide fractions of the *L. infantum* antigen was observed. No anti-*Leishmania* antibodies were detected in any of the three animals by ELISA, IFAT and WB.

Regarding the cellular immune response, a delayed type hypersensitivity (DTH) reaction to leishmanin was evaluated using an inactivated suspension of 3 × 108 L. *infantum* promastigotes (MHOM/FR/78/LEM75) per ml in 0.2% phenol-saline, with a protein content of 30 µg/ml. The solution (100 µl) was intradermally injected in the skin of the groin. Skin reactions were recorded after 72 h and an induration or erythematous area > 5 mm in diameter was considered positive [24]. In these three patients, the DTH reaction to leishmanin was negative, and no induration or erythematous area was observed prior to anti-*Leishmania* treatment.

The identification of the parasite within lesions in the affected organs clearly established a cause-and-effect relationship. Accordingly, anti-*Leishmania* treatment was administered, including allopurinol (10 mg/kg orally twice a day) and meglumine antimoniate (50 mg/kg subcutaneously twice a day for four weeks) for case studies 1 and 3. A good clinical response was observed, with a normal abdominal ultrasound and a negative PCR result from material obtained via ultrasound-guided splenic puncture in case 1 and the absence of dermatological signs in case 3 after meglumine antimoniate administration. In case 2, it was not possible to conduct clinical follow-up, administer anti-*Leishmania* treatment, or perform the DTH test after treatment. Finally, cell-mediated immunity, as evaluated by the DTH test, was positive in cases 1 and 3 following the administration of meglumine antimoniate. A negative DTH test result is commonly observed in dogs with clinical leishmaniosis [25], whereas a positive result is typically seen in dogs showing clinical improvement following anti-*Leishmania* treatment [26].

## Discussion and conclusions

All three dogs included in this case series were seronegative, however the presence of *Leishmania* amastigotes was confirmed by other direct methods such as cytological-histological examination alone or in combination with IHC technique. In certain cases, the parasitic load detected by histology may be low, yet the histopathological pattern remains consistent with the presence of the parasite. In this context, IHC serves as a valuable complementary technique, particularly due to the limitations of conventional haematoxylin-eosin staining in reliably detecting amastigotes.

Other diagnostic methods have been described in the literature. Ultrasound-guided splenic puncture can provide material for confirming *L. infantum* infection through cytology, PCR or parasite culture [27, 28]. Additionally, biopsies of cutaneous lesions or the spleen can also be utilized, as demonstrated in cases where cytological and histological examinations were performed, either alone or in combination with IHC.

The detection of dogs with clinical leishmaniosis and null-specific immune response against *L. infantum* is uncommon and these circumstances should be taken into account from a diagnostic standpoint in the future. The production of specific antibodies has been found to be low in initial and late phases of infection in dogs with clinical leishmaniosis [9]. In one study, a dog with clinical signs of leishmaniosis and with parasitological confirmation, was seronegative by direct agglutination test and WB [29]. The authors of the article did not discuss the potential reasons for this unexpected finding. In human leishmaniasis, the absence of humoral response has been described in children in Spain. From a total of 14 cases of visceral leishmaniasis, 11 patients were seropositive by IFAT, whilst the remaining cases were seronegative [30].

Nowadays, there are two clinical staging systems of CanL available to classify patients according to the severity of their clinical presentation, the LeishVet staging system [3] and the Canine Leishmaniasis Working Group (CLWG) classification system [2]. According to LeishVet, the level of anti-*Leishmania* antibodies is classified from a negative to low positive result, from a low to high positive result or medium to high positive antibody levels in combination with clinical signs and laboratory findings to determine the clinical stage of the patient. By contrast, CLWG staging classification is based on the combination of several diagnostic method results including quantitative serology and direct detection methods together with clinical signs and laboratory findings. In this sense, it is possible to detect exposed dogs with low positive antibody levels and negative cytology, histology or PCR, infected dogs with low positive antibody levels with positive cytology, histology or PCR, and sick dogs with high positive antibody levels or low level with positive cytology, histology or PCR. Based on this case series, these three patients would be unclassifiable with both the LeishVet staging system and CLWG, due to the negative results found in serology.

In general, detection and quantification of antibody levels is considered a key diagnostic technique [3, 12, 31]. Moreover, variations in antibody levels have been considered essential for monitoring cases during and after treatment of CanL. A decrease in antibody levels has been associated with clinical improvement or by contrast, an increase in antibody levels interpreted as a disease relapse [2]. Specific immune response by the dog against the parasite is dependent on the individual´s immune reactivity, being highly variable among dogs [32]. Asymptomatic infected dogs can be seronegative, possibly due to an effective cellular immunity [32–34]. Additionally, dogs with chronic *Leishmania*-induced cutaneous lesions have been presented with absence or low levels or *Leishmania*-antibodies [17]. In our study one dog with *Leishmania*-induced cutaneous lesions and two dogs with systemic leishmaniosis presented null levels of anti-*Leishmania* antibodies.

The null or low titre of anti-*Leishmania* antibodies could be correlated with the large span between infection and seroconversion (3 months to 7 years) in asymptomatic animals [35]. However, dogs with clinical leishmaniosis generally present with high titres of anti-*Leishmania* antibodies [36]. Other parasite infections like the ones mediated by *Plasmodium* spp. or *Schistosoma haematobium*, are capable of inducing a decrease in B-cells, production of low-affinity antibodies and the suppression of a specific antibody response [37–39]. Additionally, *Leishmania major* has been shown to generate immunosuppressive regulatory B cells by means of secretory factors or antigens [40, 41]. Up until now a marked humoral response has been suggested to favour the infection of *Leishmania* and increase its pathogenicity [42, 43]. However, the role of B-cells in CanL is controversial [44].

It is important to consider the value of case follow-up and the implementation of additional diagnostic techniques as described in the literature. Material obtained through ultrasound-guided splenic puncture can confirm *L. infantum* infection via PCR, parasite culture or cytology [19, 27, 28]. Additionally, biopsies of cutaneous lesions or the spleen can be utilized, as demonstrated in cases where cytological and histological examinations were performed, either alone or in combination with specific IHC. In a recent study, the presence of amastigotes was identified through gastrointestinal biopsies, and leishmaniosis was diagnosed using serology in 10 of 15 dogs (66.7%), serology combined with blood PCR in 3 of 15 (20.0%), lymph node cytology in 1 of 15 (6.7%), and blood PCR in 1 of 15 (6.7%) [45].

In conclusion, this series of cases suggests the presence of dogs with clinical leishmaniosis that do not present a specific-humoral response against the parasite. The combination of additional confirmatory techniques such as cytological-histopathological examination in association with IHC is strongly recommended in suspected cases.

## Data Availability

The datasets used and/or analysed during the current study are available from the corresponding author on reasonable request.

## Declarations

### Prior publication

Data have not been published previously.
